# Identification of a novel *Shank2* transcriptional variant in *Shank2* knockout mouse model of autism spectrum disorder

**DOI:** 10.1186/s13041-020-00595-4

**Published:** 2020-04-06

**Authors:** Yong-Seok Lee, Nam-Kyung Yu, Jeewan Chun, Jung-eun Yang, Chae-Seok Lim, Hyopil Kim, Gaeun Park, Jin-A Lee, Kyungmin Lee, Bong-Kiun Kaang, Jae-Hyung Lee

**Affiliations:** 1 Department of Physiology, Biomedical Sciences, Neuroscience Research Institute, Seoul National University College of Medicine, Seoul, 03080 South Korea; 2 Laboratory of Neurobiology, School of Biological Sciences, College of Natural Sciences, Seoul National University, Seoul, 08826 South Korea; 3 Department of Dentistry, Graduate School, Kyung Hee University, Seoul, 02447 South Korea; 4 Department of Pharmacology, Wonkwang University School of Medicine, Iksan, 54538 South Korea; 5 Department of Biotechnology and Biological Sciences, Hannam University, Daejeon, 34430 South Korea; 6 Behavioral Neural Circuitry and Physiology Laboratory, Department of Anatomy, Brain Science & Engineering Institute, Kyungpook National University Graduate School of Medicine, Daegu, 41944 South Korea; 7 Department of Life and Nanopharmaceutical Sciences, Department of Oral Microbiology, School of Dentistry, Kyung Hee University, Seoul, 02447 South Korea

**Keywords:** Autism spectrum disorder, RNA-seq, *Shank2*

## Abstract

Autism spectrum disorder (ASD) is a group of neurodevelopmental disorders that are highly heterogeneous in clinical symptoms as well as etiologies. Mutations in *SHANK2* are associated with ASD and accordingly, *Shank2* knockout mouse shows ASD-like behavioral phenotypes, including social deficits. Intriguingly, two lines of *Shank2* knockout (KO) mouse generated by deleting different exons (exon 6–7 or exon 7) showed distinct cellular phenotypes. Previously, we compared gene expressions between *Shank2* KOs lacking exon 6–7 (e6–7 KO) and KOs lacking exon 7 (e7 KO) by performing RNA-seq. In this study, we expanded transcriptomic analyses to identify novel transcriptional variants in the KO mice. We found prominent expression of a novel exon (exon 4′ or e4’) between the existing exons 4 and 5 in the *Shank2* e6–7 KO model. Expression of the transcriptional variant harboring this novel exon was confirmed by RT-PCR and western blotting. These findings suggest that the novel variant may function as a modifier gene, which contributes to the differences between the two *Shank2* mutant lines. Furthermore, our result further represents an example of genetic compensation that may lead to phenotypic heterogeneity among ASD patients with mutations in the same gene.

## Introduction

Autism spectrum disorder (ASD) is a group of neurodevelopmental disorders that are diagnosed by impairments in social–communication and restricted, repetitive behaviors or interests [1]. However, ASD is highly heterogeneous in terms of clinical symptoms and etiology. Although ASDs are highly heritable, and recent advances in next generation sequencing have revealed more than 1000 mutations associated with ASD, most of those identified alleles are rare variants that represent only a small fraction of ASD [2]. Moreover, it is becoming clear that ASD involves multigenic interactions, as well as interactions between risk genes and various environmental factors, which further highlight its complexity [2, 3].

Mutations in *SHANK* genes are associated with ASD and other psychiatric conditions such as intellectual disabilities, schizophrenia, and Phelan-Mcdermid Syndrome [4, 5]. *SHANK* genes (*SHANK*1, 2, 3) encode the SH3 and multiple ankyrin repeat domain proteins, which are post-synaptic scaffold proteins of excitatory synapses [6, 7]. Among *SHANK* genes, over 80 and 30 ASD-associated mutations have been identified in *SHANK3* and *SHANK2*, respectively (https://gene.sfari.org/database/human-gene/) [8, 9]. While Shank3 mutant mice have been most extensively characterized, previous studies demonstrated that *Shank2* null mutant mice also exhibit ASD-like behavioral deficits and synaptic dysfunctions [7, 10–12]. Interestingly, *Shank2* KO mice lacking exons 6 and 7 (*Shank2* e6–7 KO) showed hypo-function of *N*-methyl-D-aspartate receptor (NMDAR) and impaired long-term potentiation (LTP), whereas the other *Shank2* mutant lacking exon 7 of the *Shank2* gene (e7 KO) showed hyper-function of NMDAR and enhanced LTP in the adult hippocampus [10, 11]. Recent studies showed that differences in gene expressions, experimental conditions, genetic background, and the developmental stages of the mice may, at least partially, explain the opposite cellular phenotypes in the two *Shank2* mutant mouse models [13–15]. We have previously compared the gene expression profiles of *Shank2* e6–7 and e7 KO mice by performing RNA-seq analyses, and found that the two KO mouse lines share only a few differentially expressed genes [14]. In this study, we analyzed RNA-seq data from the hippocampi of two *Shank2* mutant mouse models (e7 and e6–7 KO), and found a novel transcript that encodes a new exon in e6–7 and e7 KO mice. We confirmed the expression of this novel transcript at both mRNA and protein levels, which suggested that this novel variant may act as a modifier gene that contributes to differences between the two *Shank2* mouse mutant lines.

## Materials and methods

### Bioinformatics

The RNA-Seq raw reads [GSE79824 (GSM2104342 - GSM2104353) and GSE47966] were processed and mapped against the mouse genome, as previously described [14]. The uniquely mapped reads were used to reconstruct *Shank2* gene transcripts (guided transcriptome reconstruction), as previously described [16]. The transcribed regions based on the mapped RNA-Seq reads were analyzed to detect possible novel exons in the known gene. The spliced junction reads between a known exon and a novel exon were used to confirm the expression of the novel exon and to reconstruct the transcript isoforms. Conserved regions on the novel exon were evaluated using the comparative genomics tracks in the UCSC genome browser. Multiple sequence alignments among eight different species (human, rhesus, mouse, rat, dog, cow, chicken and frog) was performed using the MUSCLE algorithm implemented in EMBL-EBI bioinformatics tools [17]. Based on the multiple sequence alignment result, a phylogenetic tree was constructed using the neighbor-joining method implemented in MEGA [18]. Methylation information in the mouse genome was obtained from the “DNA Methylation” track hub in the UCSC genome browser provided by MethBase [19].

### Quantitative real time polymerase chain reaction (PCR)

Total RNA samples were extracted from the prefrontal cortex, hippocampus, and cerebellar hemisphere region of 4-week and 8-week old male C57BL/6 N mice (Orient Bio). cDNA was extracted from total RNA by reverse-transcription using SuperScript III (Invitrogen) for RNA-seq. Primer sequences used were as follows: forward 5′-CGGTCTGAGTGAGATGGTCA-3′ (exon 4′) and reverse 5′-TAGGAGCCCACCGTGTAATG-3′ (exon 5). Quantitative RT-PCR was performed with SYBR Premix Ex Taq II (Tli RNase H Plus) (Takara) on the ABI7300 instrument. Initial activation was conducted at 95 °C for 30 s. This was followed by 40 cycles of 95 °C for 5 s and 60 °C for 30 s. Single peaks from dissociation cycles were confirmed, which were indicative of a single PCR product. The 2^-ΔΔCt^ method was used for quantitative comparison, using GAPDH as the control. RT-PCR was performed using Kapa HiFi Hotstart Readymix (Kapa Biosystems). Initial activation at 94 °C was carried out for 30 s, and was followed by 34 cycles of 94 °C for 30 s and 58 °C for 30 s (*Actb* and ex4’f-ex5r), or 60 °C for 30 s (ex2f-ex4r and ex4f-ex6r) and 72 °C for 60 s. The RT-PCR products were visualized in agarose gels.

### Western blot

Western blot analyses were performed as previously described [14, 20]. Membranes were incubated with a rabbit anti-SHANK2 (ab171189, Abcam) or anti-tubulin (T4026, Sigma) antibodies overnight at 4 °C, followed by incubation with an appropriate HRP-conjugated secondary antibody for 2 h at room temperature. Chemiluminescence signals were acquired and analyzed using ChemiDoc (Bio-Rad).

## Results

### Identification and validation of a novel transcript

**Fig. 1:**
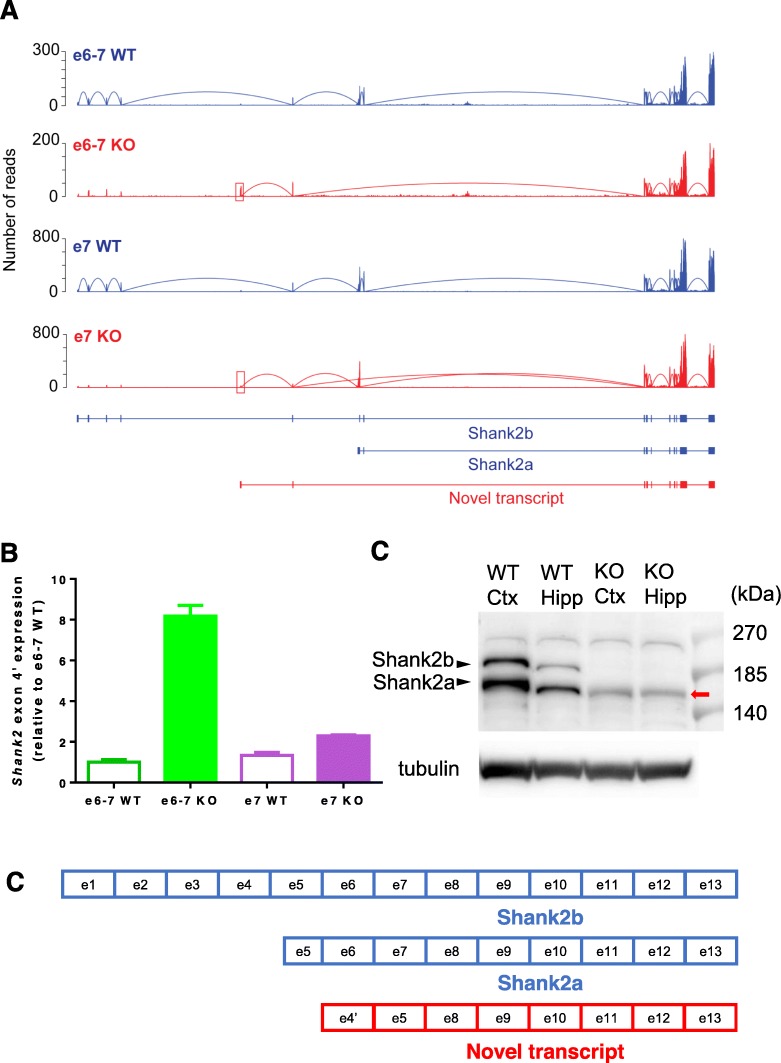
Identification of the novel exon 4′ in *Shank2* KO mice and validation of mRNA and protein expression of this transcript. **a** Read distribution plots and transcript structures in e6–7 and e7 knockout (KO) and their own wild type (WT) littermate samples. RNA-Seq data (NCBI GEO GSE79824; GSM2104342 - GSM2104353) were processed as described in the method section. Blue color represents WT samples and red color represents KO samples. Red box shows the identified novel exon 4′ in KO samples. **b** Real time quantitative PCR using primers that span exon 4′ and 5. Five hippocampi per group were used for the analysis; experiments were carried out in triplicates. **c** A schematic exonic diagram of translated proteins from the different Shank2 transcripts. **d** Western blot analysis of the Shank2 proteins. The long (Shank2b) and short (Shank2a) Shank2 isoforms were detected in WT, which were completely missing in e6–7 KO. In e6–7 KO, a clear band for the novel isoform (red arrow) was detected slightly below the band for Shank2a. The difference between the two bands are emphasized in a different electrophoresis condition (Fig. S1)

We extended our comparative transcriptome analysis by measuring expression densities through the transcripts, including intronic regions. Previous studies have identified multiple splicing variants of *Shank2*, including short *Shank2a* and long *Shank2b* [21, 22]. In this study, we also identified a previously unknown intronic region via RNA sequencing mapping, which suggests that there may be an additional *Shank2* splicing variant in the mouse hippocampus (Fig. 1a). To characterize the region in more detail and determine whether the identified expression region was a true transcribed region, we reconstructed KO and wild type (WT) *Shank2* transcripts based on the RNA raw sequencing reads (Fig. 1a). The reads from the spliced junction between the known exons and newly identified transcribed regions were used to confirm the expression of the novel exon. We were able to reconstruct *Shank2* transcripts in each sample (e6–7 KO, e6–7 WT littermate, e7 KO, and e7 WT littermate), and confirmed the previously known transcripts (Fig. 1a). We identified the transcribed regions, chr7:144238708–144,239,093 (e6–7 KO) and chr7:144238699–144,239,093 (e7 KO) (red box in Fig. 1a), which were located in exons 4 and 5 of the *Shank2* transcript in the reconstructed e6–7 and e7 KO transcripts. This novel transcribed region has not been previously annotated in any gene annotation database, and we annotated the novel transcribed region as exon 4′. We found that the novel exon 4′ is connected with exon 5, but not with the upstream exons; therefore, exon 4′ is the first exon of this novel transcript. More interestingly, although expression of the novel transcript containing exon 4′ was detected in both e6–7 and e7 KO, but very weakly in e7 KO (Fig. 1a).

To verify the expression of the novel *Shank2* transcript containing the newly discovered exon 4′, we designed PCR primers with the forward primer in exon 4′ (ex4’f) and the reverse primer in exon 5 (ex5r) (Table 1). Quantitative RT-PCR detected a single PCR product, confirming the existence of the exon 4′-5 containing transcript. Expression of the novel transcript was markedly increased by approximately 8 folds in the e6–7 KO compared with that of e6–7 WT littermate (Fig. 1b). However, consistent with the RNA-Seq results, expression of the novel transcript was considerably less increased in e7 KO (~ 1.71 fold compared with that of ex7 WT littermate, Fig. 1b). To further confirm that the novel transcript is translated into a protein of the expected size in the brains of e6–7 KO mice (Fig. 1c), we performed western blot analyses. A clear protein band was detected slightly below the band for Shank2a only from the cortex and hippocampus of e6–7 KO mice (Fig. 1d and Fig. S1). Both long (Shank2b) and short (Shank2a) Shank2 isoforms were not detected in e6–7 KO mice, which confirmed the specificity of the antibody (Fig. 1d and Fig. S1). Although expression of the novel transcript was also increased in e7 KO, it would be difficult to be detected in western blot due to its relatively lower expression level compared with that in e6–7 KO.

**Table 1:** List of primers for quantitative RT-PCR

Name	Sequence
ex2f	CTTCACTCAACAGGCTGGGTG
ex4f	TGGTTCCCAGCTGAGTGTGTG
ex4’f	CGGTCTGAGTGAGATGGTCA
ex4r	CTCTCCGATGCTCAGAACTTTGAC
ex5r	TAGGAGCCCACCGTGTAATG
ex6r	CCAAAGCCCTCGTTGTCCTTC
*Actb* forward	CTTCTCCAGGGAGGAAGAGG
*Actb* reverse	AGCCATGTACGTAGCCATCC

### Characterization of the novel transcript

**Fig. 2:**
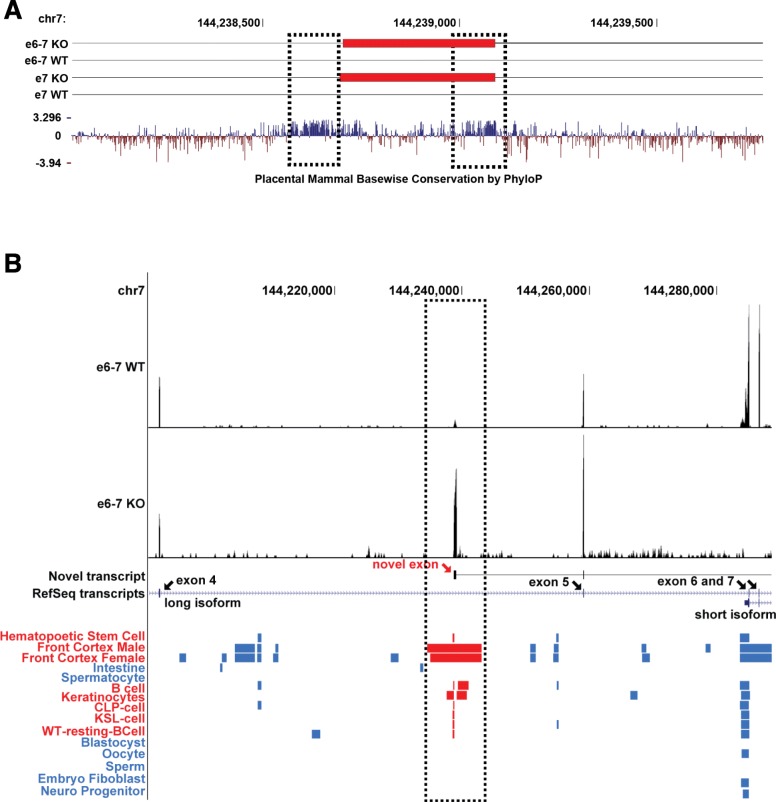
Genomic landscape of the novel exon 4′ in *Shank2*. **a** UCSC genome browser conservation track (Placental Cons) for regions around exon 4′. Dotted black boxes indicate highly conserved regions. **b** UCSC DNA methylation tracks. DNA methylation changes are shown as blue or red (near exon 4′) boxes. Red boxes represent the regions near the identified novel exon. Black arrows indicate known exons in the *Shank2* transcript. Red arrow indicates exon 4′

Unexpectedly, when we analyzed phylogenetic conservation of exon 4′ in the UCSC genome browser using comparative genomics tracks (conservation, Placentral Chain/Net, and Vertebrate Chain/Net), we found that it is well-conserved across vertebrate animals, except for fish species. As shown in Fig. 2a, there are two highly conserved regions. One of the conserved regions is located upstream of exon 4′ (left dotted black box in Fig. 2a), which could serve as the promoter for its expression. The other highly conserved region is located at the end of exon 4′ (right dotted black box in Fig. 2a), which could be the protein coding region in the novel transcript. We extracted DNA sequence that spans across the novel exon 4′ sequence, as well as 200 bp upstream of the exon 4′ sequence from eight different representative vertebrate species (except birds). Multiple sequence alignment and phylogenetic analyses were conducted. Expectedly, the overall sequence was well-aligned among the species, and the phylogenetic tree among the eight different species revealed that no evidence showing rapid evolutionary rate change was observed (Fig. S2).

We assessed the methylation profiles of regions near exon 4′ in several tissues using the DNA methylation hub track in the UCSC genome browser. Interestingly, there are overlaps between the exon 4′ region and hypomethylated regions in several tissues (Fig. 2b). Especially, the hypomethylated regions in brain tissues are associated with the tissue-specific epigenetic reconfigurations during mammalian brain development [23]. Therefore, the novel exon 4′ is not only evolutionary conserved, but may also play functional roles in the brain.

### Expression of novel transcripts in wild type mouse brain

**Fig. 3:**
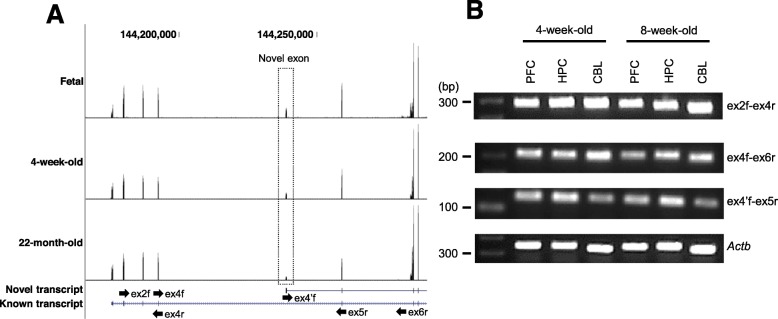
Expression of the novel transcripts in wild type mouse brain regions. **a** Read distribution plots and transcript structures in fetal, 4-week-old, and 22-month old mouse RNA-Seq samples. The dotted box highlights the novel exon. Black arrows represent the RT-PCR primers used to validate the expression of the known and novel transcripts in mouse brains. **b** Representative RT-PCR of 4-week-old and 8-week-old mouse brains regions (prefrontal cortex, PFC; hippocampus, HPC; and cerebellum, CBL)

To determine whether the novel transcript is constitutively expressed in wild type mouse brains, we investigated the public brain transcriptome data from the study by Lister et al. [23]. They determined the temporal gene expression profiles of the mouse frontal cortex from fetal to 22-month-old mice using RNA sequencing. We collected and analyzed the RNA-Seq data for the following three data time points: fetal, 4-week-old, and 22-month-old. After constructing the *Shank2* gene isoforms, as previously described, we confirmed that the novel transcripts were expressed in all time points (Fig. 3a). Furthermore, we performed RT-PCR using extracted RNAs from 4-week-old and 8-week-old wild type C57Bl/6 N mouse brain regions (prefrontal cortex, PFC; hippocampus, HPC; and cerebellum, CBL). Three different primer pairs were used to detect the known and novel transcripts (ex2f-ex4r, ex4f-ex6r: known transcript; ex4’f-ex5r: novel transcript) (Fig. 3a black arrows). As shown in Fig. 3b, we confirmed that both known and novel transcripts were expressed in each brain region.

## Discussion

We occasionally observe conflicting, heterogeneous phenotypes in the same genetic mutant lines associated with ASD. In addition to the two *Shank2* KO lines studied here, mutant mouse lines of another *Shank* isoform, in which either exons 4–9 or exons 4–7 of *Shank3* have been deleted, showing different synaptic and behavioral phenotypes when compared to each other [24–26]. *Epac2* KO mice, another ASD mouse model that lacks either exon 1 or 3 also shows distinct behavioral and electrophysiological phenotypes [27–29]. However, the molecular mechanism underlying the different phenotypes in same gene mutant lines still remains unclear. In the previous study [14], we profiled whole transcriptome expressions for *Shank2* e6–7 KO and e7 KO mouse models and found that expression of the *Gabra2* gene was reduced in e6–7 KO mice, but not in e7 KO mice when compared to their own WT littermates; this may partially contribute to differences between *Shank2* e6–7 KO and e7 KO mice.

In this study, we identified a novel *Shank2* transcript, which has not been previously reported. It is rather surprising that the novel exon 4′ has not been annotated yet considering that we found that it is well-conserved across vertebrates. Relatively lower expression level of the transcript harboring the exon 4′ compared to the previously identified isoforms may have hindered the identification of this novel transcript. To validate this novel transcribed region, we computationally reconstructed transcript isoforms and experimentally confirmed the expression of the identified region using quantitative RT-PCR. In the reconstructed e6–7 KO and e7 KO transcripts, the novel transcribed region (novel exon 4′) was connected to the downstream exon 5. However, we were unable to detect the reconstructed transcripts containing the novel exon 4′ in WT samples because the RNA sequencing (RNA-Seq) depth was not enough. Furthermore, as shown in the RNA-Seq expression profile of the transcripts, expression level of the novel exon 4′ in e6–7 KO mice was greater than e7 KO mice, as determined by quantitative RT-PCR (Fig. 1b). This aberrant increase in exon 4′ expression in e6–7 KO mice may also contribute to the observed differences in the two *Shank*2 KO mutants, which remains to be examined. It would be interesting to examine whether ectopic expression of the cloned novel isoform in either WT or e7 KO mice can recapitulate, at least, some phenotypes in e6–7 KO.

Gene regulation is a complicated process and is finely tuned in different settings. Many studies have shown that epigenetic profiles such as methylation can affect differential isoform expressions [30]. For example, intragenic DNA methylome map of the brain has shown that tissue-specific DNA methylation in the *Shank3* gene regulates promoter activity, and that differential DNA methylation patterns are associated with alternative transcript expressions in tissue- and cell type-specific manners in the brain regions [31]. Our study showed that regions near the novel exon 4′ were phylogenetically well-conserved across vertebrate species and were hypomethylated in several mouse tissues, including the brain tissues. We speculate that the deletion of exon 6 and 7 may have affected the methylation status of the region near the novel exon 4′, which subsequently increases the expression of the novel *Shank2* transcript. Alternatively, other epigenetic modifications or chromatin reorganization, such as changes in nucleosome and positioning may have occurred in e6–7 KO. Further analyses would be required to test the suggested hypothesis.

## Supplementary information

**Additional file 1: Figure S1.** Western blot analysis of the Shank2 proteins. The antibody used for the analysis was same with the analysis presented in Fig. 1c. The amount of proteins loading was different and the running time was increased. **Figure S2.** Multiple sequence alignment and phylogenetic analysis of DNA sequences containing exon 4′ from eight vertebrate species. The novel exon 4′ region was highlighted by red boxes.

## Data Availability

All data generated or analyzed during this study are included in this published article or are available from GEO DataSets (https://www.ncbi.nlm.nih.gov/gds).

## Abbreviations

ASD: Autism spectrum disorder
KO: Knockout
LTP: Long-term potentiation
NMDAR: *N*-methyl-D-aspartate receptor
RT-PCR: Real time-polymerase chain reaction
WT: Wild type
