# Supplementary material for: Identification of a novel Shank2 transcriptional variant in Shank2 knockout mouse model of autism spectrum disorder
Source: Mol Brain. 2020 Apr 6;13:54. doi: 10.1186/s13041-020-00595-4 (PMC7132969; doi:10.1186/s13041-020-00595-4)
Supplement: Supplementary file 1 — Additional file 1: Figure S1. Western blot analysis of the Shank2 proteins. The antibody used for the analysis was same with the analysis presented in Fig. 1c. The amount of proteins loading was different and the running time was increased. Figure S2. Multiple sequence alignment and phylogenetic analysis of DNA sequences containing exon 4′ from eight vertebrate species. The novel exon 4′ region was highlighted by red boxes. [file 13041_2020_595_MOESM1_ESM.pptx]

## Slide 1
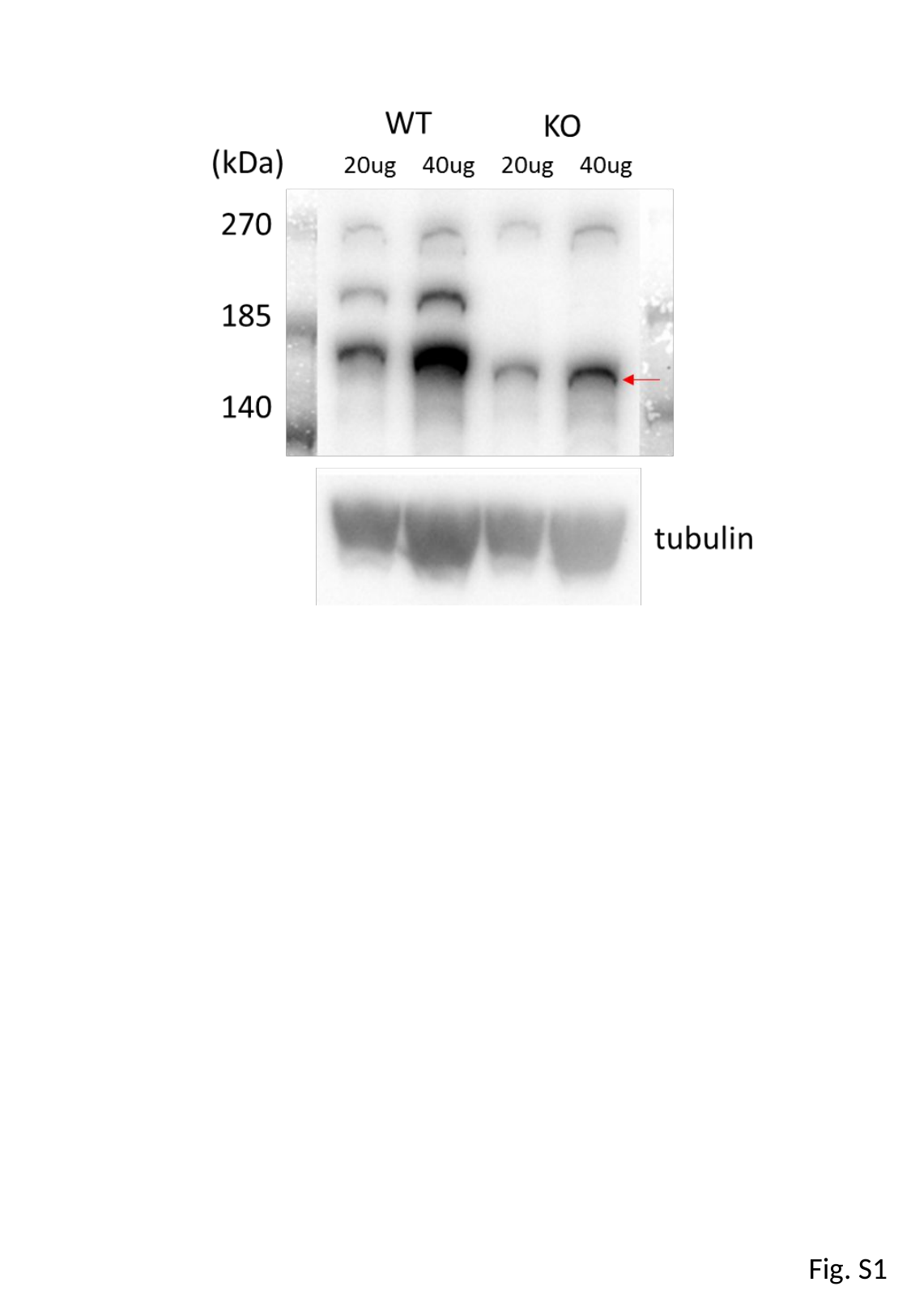

Fig. S1

## Slide 2
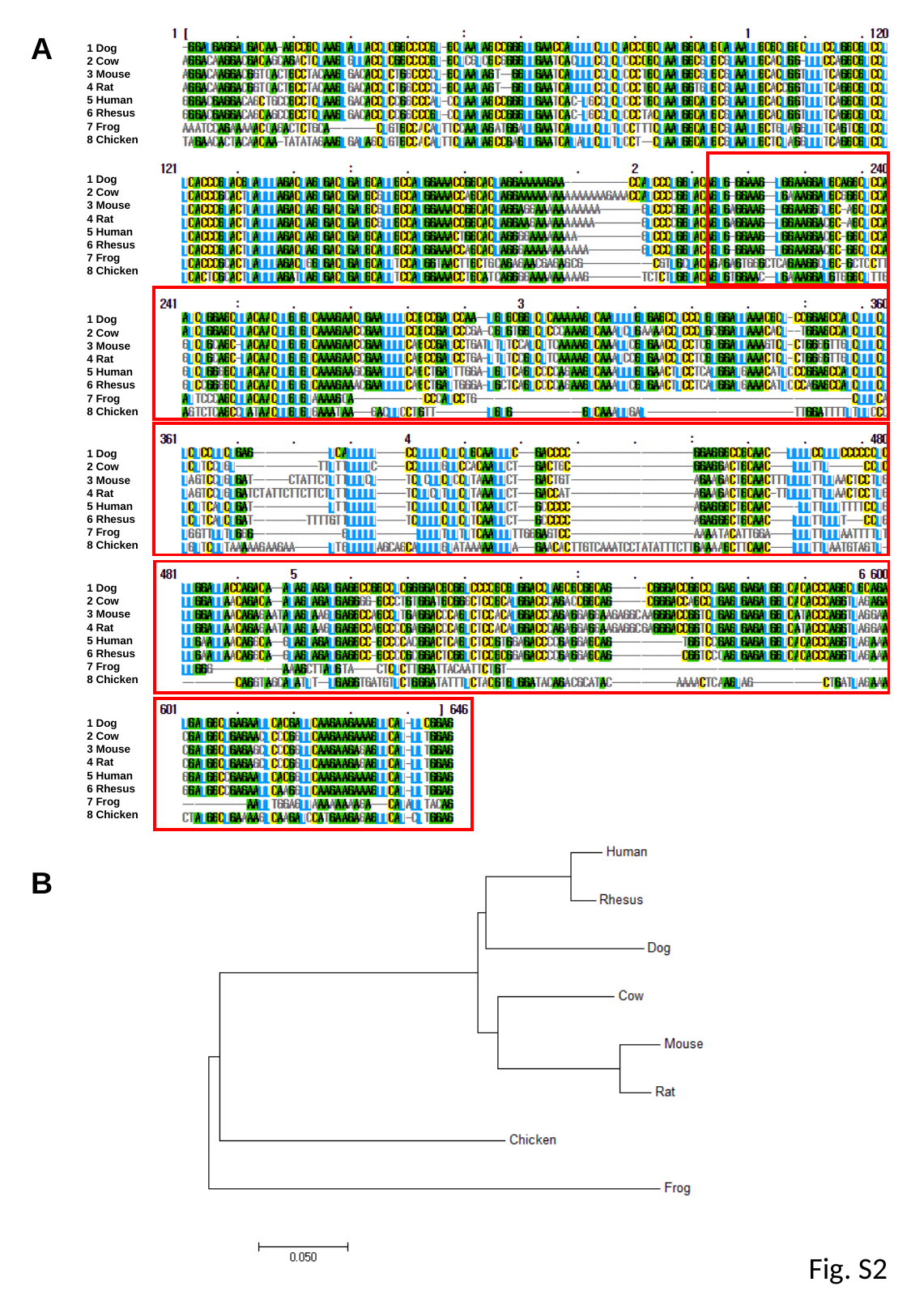

A
1 Dog
2 Cow
3 Mouse
4 Rat
5 Human
6 Rhesus
7 Frog
8 Chicken
1 Dog
2 Cow
3 Mouse
4 Rat
5 Human
6 Rhesus
7 Frog
8 Chicken
1 Dog
2 Cow
3 Mouse
4 Rat
5 Human
6 Rhesus
7 Frog
8 Chicken
1 Dog
2 Cow
3 Mouse
4 Rat
5 Human
6 Rhesus
7 Frog
8 Chicken
1 Dog
2 Cow
3 Mouse
4 Rat
5 Human
6 Rhesus
7 Frog
8 Chicken
1 Dog
2 Cow
3 Mouse
4 Rat
5 Human
6 Rhesus
7 Frog
8 Chicken
B
Fig. S2
